# Trimethylamine-N-oxide (TMAO) mediates the crosstalk between the gut microbiota and hepatic vascular niche to alleviate liver fibrosis in nonalcoholic steatohepatitis

**DOI:** 10.3389/fimmu.2022.964477

**Published:** 2022-08-22

**Authors:** Dengcheng Zhou, Jing Zhang, Chengju Xiao, Chunheng Mo, Bi-Sen Ding

**Affiliations:** ^1^ Key Laboratory of Birth Defects and Related Diseases of Women and Children of MOE, State Key Laboratory of Biotherapy, West China Second University Hospital, Sichuan University, Chengdu, China; ^2^ Fibrosis Research Program, Division of Pulmonary and Critical Care Medicine, Division of Liver Diseases, Icahn School of Medicine at Mount Sinai, New York, NY, United States; ^3^ Division of Regenerative Medicine, Weill Cornell Medicine, New York, NY, United States

**Keywords:** gut microbiota, trimethylamine N-oxide, NASH, endothelial cells, ATP1B1

## Abstract

Liver fibrosis is one main histological characteristic of nonalcoholic steatohepatitis (NASH), a disease paralleling a worldwide surge in metabolic syndromes with no approved therapies. The role of the gut microbiota in NASH pathogenesis has not been thoroughly illustrated, especially how the gut microbiota derives metabolites to influence the distal liver in NASH. Here, we performed 16S rDNA amplicon sequencing analysis of feces from a mouse NASH model induced by a Western diet and CCl_4_ injury and found genera under *Streptococcaceae*, *Alcaligenaceae*, *Oscillibacter*, and *Pseudochrobactrum*, which are related metabolites of TMAO. Injection of the gut microbial metabolite TMAO reduced the progression of liver fibrosis in the mouse NASH model. Further analysis revealed that the anti-fibrotic TMAO normalized gut microbiota diversity and preserved liver sinusoidal endothelial cell integrity by inhibiting endothelial beta 1-subunit of Na (+), K (+)-ATPase (ATP1B1) expression. Collectively, our findings suggest TMAO-mediated crosstalk between microbiota metabolites and hepatic vasculature, and perturbation of this crosstalk disrupts sinusoidal vasculature to promote liver fibrosis in NASH.

## Introduction

Nonalcoholic fatty liver disease (NAFLD) is the hepatic manifestation of cardiometabolic syndrome, which often also includes obesity, diabetes, and dyslipidemia (1, 2). A sizable minority of NAFLD patients develop NASH, which is characterized by inflammatory changes that can lead to progressive liver damage, cirrhosis, and hepatocellular carcinoma (3, 4). Recent studies have shown that in addition to genetic predisposition and diet, the gut microbiota affects hepatic carbohydrate and lipid metabolism and influences the balance between proinflammatory and anti-inflammatory effectors in the liver, thereby impacting NAFLD and its progression to NASH (5, 6).

Several studies have implicated the involvement of the gut microbiome in NASH or NAFLD in mice and humans. Microbiota from hyperglycemic or healthy mice was transferred to germ-free mice and then fed the HFD, and only mice transplanted with microbiota from hyperglycemic mice developed fasting hyperglycemia, insulinemia, and hepatic macrovesicular steatosis (7). Using 16S rDNA analysis of NAFLD-associated parameters, the abundance of bacterial species in mice fed a low-fat and high-fat diet showed an association between *Lactobacillus gasseri* and *Lactobacillus taiwanensis* and the area of lipidic droplets in the liver (8). In another study, high-fat diet-fed germ-free mice inoculated with the microbiota of NASH patients, rather than healthy donors, showed an exacerbated NASH phenotype, as manifested by increased liver steatosis and inflammation (9). A crucial function of gut microbiota is that progression toward steatohepatitis is linked to alterations in the metabolic outputs of the intestinal microbiota, including short-chain fatty acids, bile acids, phenylacetate and TMAO (10–12).

TMAO is a metabolite produced by the host in cooperation with the gut microbiota. Dietary choline and L-carnitine can serve as precursors and be degraded by gut commensal bacteria to produce trimethylamine, which is absorbed and further metabolized into TMAO by hepatic flavin-containing monooxygenase3 (13). It has been shown that plasma levels of TMAO are positively associated with the risk of adverse cardiovascular disease and renal disease in humans (14–18). In mice, TMAO feeding promotes glucose intolerance (19), thrombosis (18), cardiovascular disease (15), chronic kidney disease (20), and neurodegenerative disease (21), whereas the reduction in TMAO prevents their development. Others have pointed out that TMAO functions as a chemical chaperone (22) and could actually be beneficial. Indeed, treatment of cells with TMAO, albeit at doses that are far greater than those observed *in vivo*, can improve protein folding (23) and reduce endoplasmic reticulum (ER) stress (24). Thus, TMAO could be detrimental, beneficial, or neutral (25).

In the present study, we describe a novel and potent role of TMAO in reducing the severity of inflammation and hepatocellular damage in livers in both acute injury and NASH models. TMAO improves fibrosis during liver injury by maintaining the integrity of the endothelium and suppressing the ATP1B1 in the liver. We found that knockdown of ATP1B1 accelerated endothelial cell proliferation and angiogenesis and that ATP1B1 inhibition with digoxin reduced liver fibrosis in NASH.

Collectively, these data reveal that TMAO promotes endothelial cell proliferation and angiogenesis by inhibiting ATP1B1 expression, maintaining the integrity of blood vessels. These results identify ATP1B1 as a key molecule in NASH and provide a molecular basis and a fresh perspective for the observed effects of TMAO in antagonizing liver injury.

## Materials and methods

### Mice

C57BL/6J mice were purchased from the Model Animal Research Center of Nanjing University. All mice were housed in pathogen-free animal facilities at a constant humidity of 65 ± 15% and a temperature of 24 ± 1°C under a 12 h light/dark cycle. The animal study was approved by the Institutional Animal Care and Use Committee of West China Second University Hospital.

### Cells

Human umbilical vein endothelial cells (HUVECs) were isolated from human umbilical cords. HUVECs were cultured in an Endo GRO-VEGF Complete Culture Media Kit (SCME002, Millipore) at 37°C in a humidified atmosphere of 5% CO2. HEK293T cells were obtained from the American Type Culture Collection (ATCC). HEK293T cells were cultured in DMEM (11965092, Gibco) with 10% fetal bovine serum (FBS) (1600044, Gibco) at 37°C in a humidified atmosphere of 5% CO2.

### Liver fibrosis and NASH models

To induce liver fibrosis, eight-week-old male mice were fed a normal diet and were intraperitoneally injected with 1μL/g (1.6 g/kg) CCl_4_ every two days 9 times. The mice were randomly assigned to two groups: a CCl_4_ group and a TMAO (Sigma cat: 317594) supplemented-group. TMAO was supplemented at a dose of 75 mg/kg/day according to a previous study (26). All mice were sacrificed, and liver samples were collected two days after the last injection of CCl_4_. In addition, mice were given an intraperitoneal injection of digoxin (0.5 mg/kg) or vehicle four times a week.

Induction of the mouse NASH model. Mice were fed a HFD diet containing 21.1% fat, 41% sucrose, and 1.25% cholesterol supplemented with a fructose (23.1 g/L) and glucose (18.9 g/L) solution and were intraperitoneally injected with 0.5μL/g (0.8 g/kg) CCl_4_ every week for three months. The mice were randomly assigned to two groups: a NASH group and a TMAO-supplemented group injected with TMAO (75mg/kg). Therefore, both groups were fed the HFD either with or without TMAO. All mice were sacrificed, and liver samples were collected two days after the last injection of CCl_4_.

### Isolation of ECs

Liver tissues were washed twice with cold PBS, minced, and incubated in a digestive mixture (1 mg/ml collagenase I and 1 mg/ml dispase II in PBS) on an orbital shaker at 37°C for 20 min. Digested tissues were filtered through a cell strainer multiple times, and cells were collected at 300 × g for 5 min. After red blood cells were removed with RBC lysis buffer and washed once, hepatocytes and NPCs were separated by an additional centrifugation step at 50 g for 5 min at 4°C.

For EC (CD31+) isolation, Dynabeads sheep anti-Rat IgG (01113068, Thermo Fisher Scientific) were washed three times with 1 ml cold MAC wash buffer and incubated with CD31 (553370, BD Biosciences) antibody at 4°C for 4 hours. The beads were then washed three times with MAC wash buffer. NPCs were resuspended in 300 μl of MAC wash buffer. Two hundred microliters of Dynabeads-CD31 antibody conjugate was added to the NPC suspension and then incubated at 4°C for 30 min on a rotator. CD31+ cells were washed with cold wash buffer and then used for subsequent experiments.

### Histological analysis

The liver tissues were fixed in 4% paraformaldehyde. For histological analysis, paraformaldehyde-fixed liver tissues were embedded in paraffin, cut into 5 μm sections, and then stained with hematoxylin and eosin (H&E), Sirius Red and Masson.

### Immunofluorescence analysis

The liver tissues were embedded in OCT and stored at -80°C. OCT-embedded liver tissues were cut into 6μm sections. The sections were fixed in 4% paraformaldehyde for 15 min and then washed with PBS. Next, the sections were incubated in permeabilization solution (0.3% Triton X-100 in PBS) for 15 min and then washed with PBS. Then, the sections were incubated with anti-collagen I (ab34710, Abcam), anti-Ki67 (ab15580, Abcam) or α-SMA (ab7817, Abcam) antibodies. After washing, the sections were incubated with Alexa Fluor 488-conjugated donkey anti-rabbit IgG (711-545-152, Jackson ImmunoResearch Labs), Alexa Fluor 555-conjugated donkey anti-rabbit IgG (ab150074, Abcam) or Alexa Fluor 594-conjugated donkey anti-mouse IgG (715-585-150, Jackson ImmunoResearch Labs). The sections were washed with PBS, counterstained with 4,6-diamidino-2-phenylindole (DAPI) (10236276001, Roche) and mounted with a cover glass. The images were captured with a confocal laser microscope setup (LSM980, Zeiss) and processed using ZEN (Zeiss).

### Serum biochemistry

Blood was collected, and serum was obtained by centrifuging at 3000 rpm and 4°C for 15 min. Serum alanine aminotransferase (ALT) and aspartate aminotransferase (AST) triglyceride (TG) were detected by an Olympus AU2700 analyzer (Olympus) to reflect liver function.

### TMAO measurements

Blood was collected, and serum was obtained by centrifuging at 3000 rpm and 4°C for 15 min. Serum TMAO was measured according to the instructions of the commercial assay. TMAO Elisa Kit (gelatins, JCSW2331).

### Western blot analysis

Mouse livers or cells were lysed in RIPA lysis buffer (20-188, Millipore). The membranes were blocked with Tris-buffered saline Tween 20 containing 5% skim milk for 1 h at room temperature and then incubated with the indicated GAPDH (G3206-1OD, Servicebio), anti-collagen I, Abcam), α-SMA (ab7817, Abcam) and anti-ATP1B1 (K004215P, Solarbio) antibodies (1:1000–1:2000) overnight at 4°C. After being washed with TBST, the membranes were incubated with an HRP-linked anti-mouse IgG secondary antibody (G1214, Servicebio) or HRP-linked anti-rabbit IgG secondary antibody (G1213, Servicebio) for 1 h at room temperature.

### RNA extraction and quantitative RT–PCR

Total RNA was extracted *via* TRIzol reagent (15596026, Thermo Fisher Scientific). Total RNA (0.5-1μg) was subjected to reverse transcription with PrimeScript RT Master Mix (Takara, RR0447A). To determine the relative mRNA level, Q-PCR was performed using universal SYBR Master Mix (Q711-02, Vazyme), and gene expression was normalized to that of GAPDH. The primers used for Q-PCR are listed in **Table S1**.

### Flow cytometry

For the flow cytometric analysis of nonparenchymal cells, liver tissue was digested into a single cell, treated with RBC lysis buffer, 50 g centrifugal division NPC, and stained with antibodies FITC-CD31 (553373, BD Biosciences), PerCP-Cy™5.5-CD45 (550994, BD Biosciences), PE-F4/80 (565410, BD Biosciences), PE-Cyanine7-CD11b (25-0112-82, Thermo Fisher Scientific), and PE-Ly6G (551461, BD Biosciences). After cell fixation, flow cytometry was performed on a FACS Calibur (BD Biosciences), and the results were analyzed with Flow Jo V10.

### 16S rDNA amplicon sequencing and bacterial community analysis

Fresh colon contents (stool samples) were collected from all mice. The hypervariable region of the 16S rRNA gene (V3 + V4 [primers F341-R806]) was amplified using the KAPA HiFi Hotstart ReadyMix PCR kit (KAPA Biosystems, Boston, MA, USA). Amplicons were extracted from 2% agarose gels and purified using the AxyPrep DNA Gel Extraction Kit (Axygen Biosciences, Union City, CA, USA). The purified product was used to prepare the Illumina DNA library. Libraries were sequenced on the Illumina HiSeq PE250 platform (Illumina, San Diego, CA, USA).

### RNA-seq of mouse endothelial cells

For RNA-seq, endothelial cells (CD31+) from mouse livers were isolated as described above, and total RNA from CD31+ cells was extracted with a RNeasy Mini Kit (74104, QIAGEN). Then, a library was constructed, and RNA-seq was performed on an Illumina Nova PE150 (Illumina, America). RNA-seq data were used for follow-up analysis after quality control and reference sequence alignment.

### Cell viability

We used the CCK-8 kit to evaluate HUVEC viability. HUVECs treated with a pulsed electric field were counted, and approximately 1 × 10^4^ cells were added to the 96-well plate. Each group was replicated with three duplicate wells. Ten microliters of CCK-8 reagent (CA1210, Solarbio) were added to the 96-well plate at 6 h of cell culture, taking care not to produce bubbles. After 2 h of incubation in an incubator at 37°C, the absorbance of the sample at 450 nm was measured using Enzyme.

### Tube formation assay

Matrigel (BD Biosciences) was thawed overnight at 4°C before use and then added to 96-well plates at 60μL/well and solidified at 37°C for 30 mins. HUVECs (1×10^4^ cells/well) were resuspended in conditioned medium from sg-NC cells or sg-ATP1B1 cells and seeded in Matrigel-coated plates. Tube formation was observed under an inverted light microscope after 4 h of incubation at 37°C. The number of meshes and tubes to assess the tube formation ability of the HUVECs.

### Viral infection and transfection

LentiCRISPRv2 vectors were used to generate ATP1B1 knockout cells. HEK293 T cells were transfected by means of Lipofectamine 6000 with pSPAX2, pMD2. Gand LentiCRISPRv2 containing a guide RNA (gRNA) that targeted human ATP1B1 and NC. Lentiviruses were collected 48 h later and were applied to infect HUVECs. Subsequently, selection with puromycin (1μg/ml) was carried out.

### Transmission electron microscope

Livers were perfusion-fixed *via* the abdominal aorta with 2.5% glutaraldehyde and then stored overnight in fixative at 4°C. Liver tissues were cut into pieces (1 × 1 × 5 mm). After washing in PBS, the samples were postfixed in 1% osmium tetroxide. After dehydration, the sample was embedded in resin, and ultrathin sections were stained with uranyl acetate and lead citrate for ultrastructural examination by a transmission electron microscope.

### Scanning electron microscope

The liver tissue sections were precisely cut to a size of 1 mm^3^ and fixed overnight in 2.5% glutaraldehyde. Then, 1% osmium tetroxide was used for staining. These tissue sections were further processed by stepwise dehydration with an ethanol gradient and vacuum dried overnight. The stubs were applied with sputtered metal coatings of gold, and the observations were captured using a field emission scanning electron microscope.

### Statistical analysis

All data are expressed as the mean ± SEM. Statistical significance was determined by the unpaired two-tailed t test using GraphPad Prism 8.0 software (San Diego, CA, USA).

Differences were considered statistically significant at p < 0.05.

## Results

### 16S rDNA amplicon sequencing analysis of feces from a mouse NASH model

**Figure 1:**
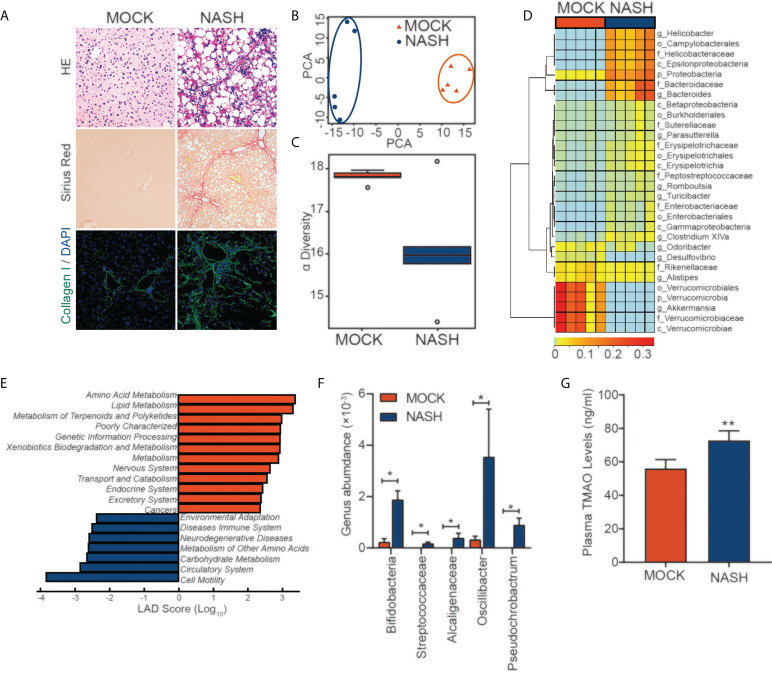
16S rDNA amplicon sequencing analysis of feces from a mouse NASH model **(A)** NASH was induced in the wild-type mice with a HFD diet and (0.8 g/kg) CCl_4_ every week for three months. Evaluation by H&E, Sirius red staining and IF for collagen (I)**(B)** PCA of the fecal microbial community between MOCK and NASH patients (n=5). **(C)** The alpha diversity of the gut microbiota (n=5). **(D)** Genus-level analysis of the relative abundances. **(E)** LEfSe (LDA effect size) analysis of KEGG pathways in MOCK and NASH. **(F)** Relative abundances of genera under *Streptococcaceae*, *Alcaligenaceae*, *Oscillibacter*, and *Pseudochrobactrum* in MOCK and NASH (n = 5). **(G)** Serum TMAO levels in the MOCK and NASH groups were quantified with a mouse TMAO ELISA Kit. *p<0.05, **p<0.01.

To better simulate the pathogenesis of NASH, we established a murine NASH model with rapid progression of extensive fibrosis by using a Western diet (WD), which is high-fat, high-fructose and high-cholesterol, combined with a low weekly dose of intraperitoneal carbon tetrachloride (CCl_4_), which serves as an accelerator (**Figure 1A**). Next, we performed 16S rDNA amplicon sequencing analysis of feces from mice with NASH. To display the microbiome space between samples, PCA indicated a symmetrical distribution of the fecal microbial community among all samples (**Figure 1B**). Compared with the control, fecal microbial diversity was decreased in NASH, as examined by alpha diversity (**Figure 1C**). LEfSe (LDA Effect Size) analysis was used to examine the effect of KEGG pathways on the differential effect of each component, and we found that changes in metabolic and immune pathways were most significant in the NASH group (**Figure 1E**). Genus-level analysis showed an increase in the relative abundances of *Streptococcaceae*, *Alcaligenaceae*, *Oscillibacter*, and *Pseudochrobactrum*, which are related metabolite TMAO (27–29), in NASH (**Figures 1D, F**). Consistent with the elevated abundance of the TMAO-producing microbiota, we found that serum TMAO levels were much higher in the NASH group than in the control group, as detected by a TMAO ELISA Kit (**Figure 1G**). These findings suggest that the gut-derived microbial metabolite TMAO may play an important role in regulating NASH.

### TMAO restores the diversity of the gut microbiota in NASH

**Figure 2:**
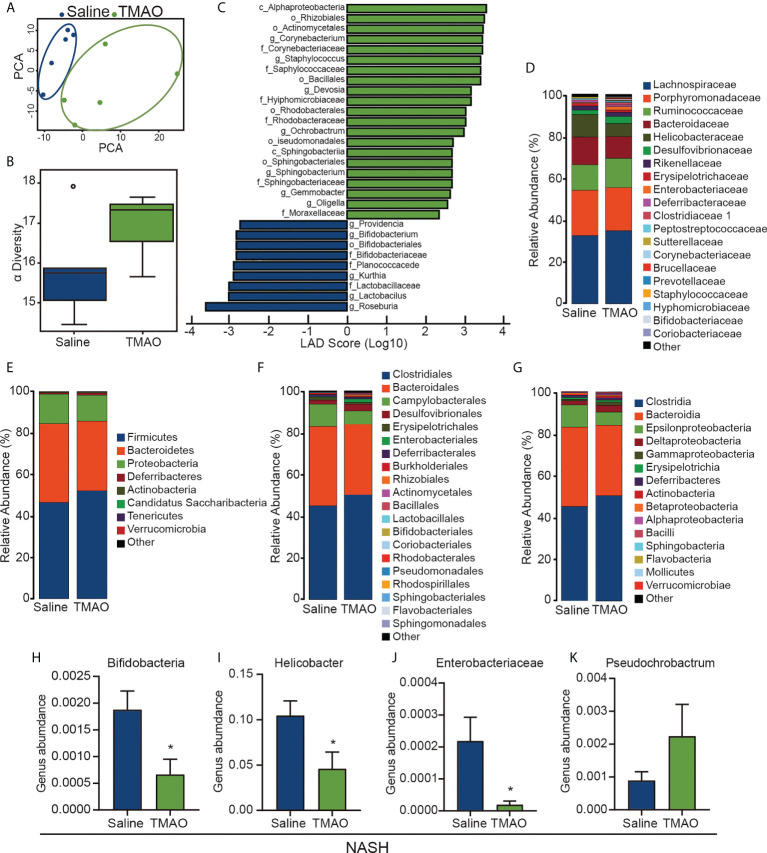
TMAO restores the diversity of the gut microbiota in NASH **(A)** PCA of the fecal microbial community among NASH and NASH-TMAO (n=5). **(B)** The alpha diversity of the gut microbiota (n=5). **(C)** Linear discriminant analysis coupled with effect size measurement analysis. **(D–H)** The differences between the two groups at the phylum, family, order and class levels. **(I–K)** Relative abundances of the genera *Pseudochrobactrum*, *Bifidobacteria*, *Helicobacter* and *Enterobacteriaceae*. *p<0.05.

Gut microbiota dysbiosis is considered to contribute to the pathogenesis of NASH. We collected fecal samples from patients with NASH and performed 16S rDNA sequencing to examine the effect of TMAO on the gut microbial profile. PCA indicated a symmetrical distribution of the fecal microbial community between the NASH-TMAO and NASH-saline groups (**Figure 2A**). We then examined the effect of TMAO on the alpha diversity of the gut microbiota. The results showed a restoration of alpha diversity with TMAO intervention in NASH (**Figure 2B**). Next, we performed linear discriminant analysis coupled with effect size measurements to discriminate the gut bacteria altered by TMAO treatment (**Figure 2C**). Meanwhile, we compared the differences between the two groups at the phylum, family, order and class levels (**Figures 2D–H**). Compared to the NASH-saline group, an increased abundance of *Pseudochrobactrum* and a decreased abundance of *Bifidobacteria*, *Helicobacter* and *Enterobacteriaceae* were observed in the NASH-TMAO group (**Figure 2I–K**). Taken together, these findings suggest that TMAO can regulate the structure of the gut microbiota and restore the depleted diversity in NASH.

### TMAO alleviates liver fibrosis in murine liver fibrosis and NASH models

**Figure 3:**
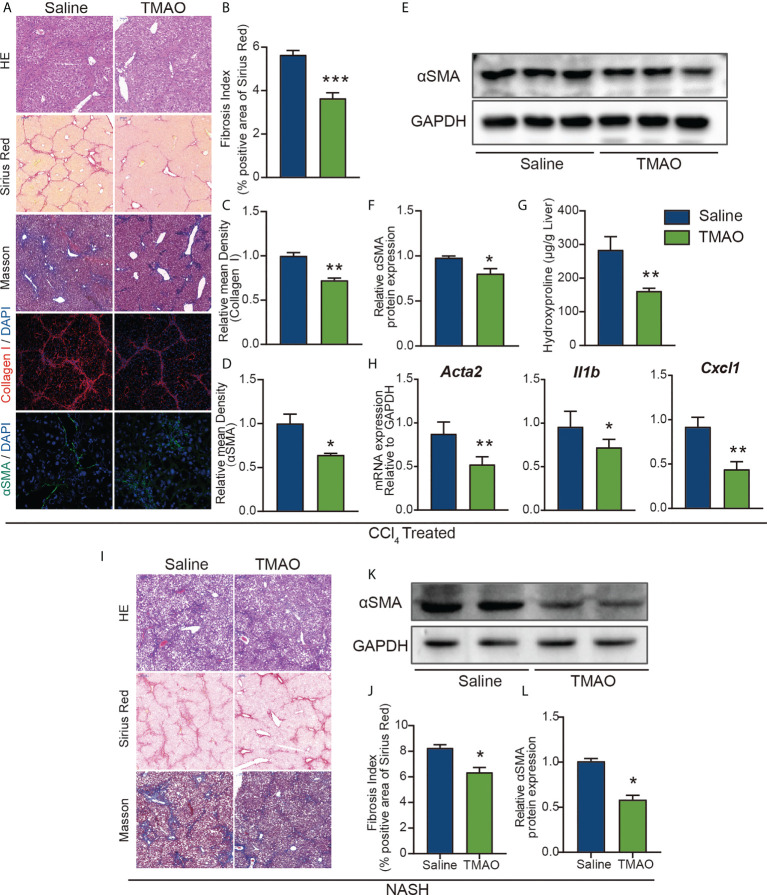
TMAO alleviates liver fibrosis in murine liver fibrosis and NASH models **(A–F)** Wild-type mice were injected with CCl_4_ every two days 9 times and normal saline or TMAO (75mg/kg) injection on the remaining days. (n=8/group) Liver fibrosis was analyzed by H&E, Sirius red, Masson staining and IF for collagen I and αSMA **(A–D)**, WB for αSMA **(E–F)**, the content of hydroxyproline **(G)**, hepatic αSMA, CXCL1 and IL1β were quantified by the RT–PCR assay **(H)**. **(I–L)** Mice were treated with WD/CCl_4_ for up to 16 weeks and normal saline or TMAO (75mg/kg) injection on the remaining days. H&E, Masson and Sirius Red staining of representative mice treated with normal saline or TMAO **(I–J)**, WB for αSMA **(K–L)**. *p<0.05, **p<0.01, ***p<0.001.

To further elucidate the effect of TMAO on liver fibrosis, we employed a chronic CCl_4_ injury model and NASH model in the study. Hepatic histological analyses of H&E, Sirius red and Masson staining demonstrated that intraperitoneal injection of TMAO alleviated liver fibrosis in the acute injury liver fibrosis model (**Figures 3A, B**). Consistently, the expression of collagen I and αSMA and the content of hydroxyproline in the TMAO-treated group were also significantly decreased compared to those in the saline-treated group, as shown by immunostaining and Western blotting (**Figures 3A–G**). Moreover, the mRNA levels of αSMA, CXCL1 and IL-1β were downregulated in the livers of TMAO-treated mice (**Figure 3H**). In addition to the acute CCL_4_ liver fibrosis model, we also found that liver fibrosis was significantly attenuated after TMAO treatment in the NASH model, as determined by H&E, Sirius red, Masson staining and Western blot of αSMA (**Figures 3I–L**). Collectively, these findings showed that TMAO supplementation ameliorated liver fibrosis in murine liver fibrosis and NASH models.

### TMAO protects the integrity of vascular endothelial cells

**Figure 4:**
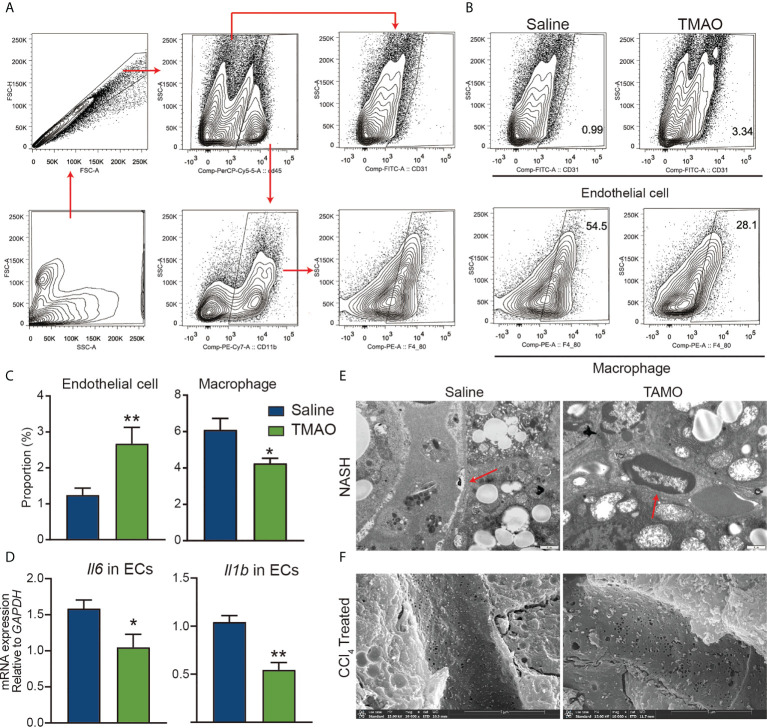
TMAO protects the integrity of vascular endothelial cells **(A–C)** Representative flow cytometry data of endothelial, macrophages and neutrophil cells in liver nonparenchymal cells (NPCs). **(D)** mRNA levels of IL-6 and IL-1β in endothelial cells of the liver. **(E)** Basement membrane (red arrows) changes were detected by TEM. **(F)** Fenestrations changes were detected by SEM. *p<0.05, **p<0.01.

Early observational work reported an association between atherosclerosis and elevated levels of TMAO (15), playing a role as modulators of vascular function. Injection of TMAO significantly increased the number of endothelial cells and decreased the number of M1 macrophages (**Figures 4A–C**). Moreover, the mRNA levels of IL-6 and IL-1β in isolated liver endothelial cells of TMAO-treated mice were significantly lower than those of saline-treated mice (**Figure 4D**). The increase in the basement membrane is one of the important factors in the occurrence of liver fibrosis (30). TEM analysis revealed that the basement membrane was reduced in vascular of TMAO-treated mice (**Figure 4E**). There is growing evidence that fenestrations may work as a permselective ultrafiltration installation, which is important for the hepatic uptake of substrates, particularly the metabolism of lipoproteins. Aberrant fenestrated structure has been considered a vital factor in liver lipid metabolism disorders (31, 32). SEM analysis revealed that fenestrations in liver sinusoidal endothelial cells (LSECs) were increased in TMAO-treated mice (**Figure 4F**). Our findings demonstrate that injection of TMAO protects murine vascular function.

### TMAO inhibits ATP1B1 expression in vascular endothelial cells

**Figure 5:**
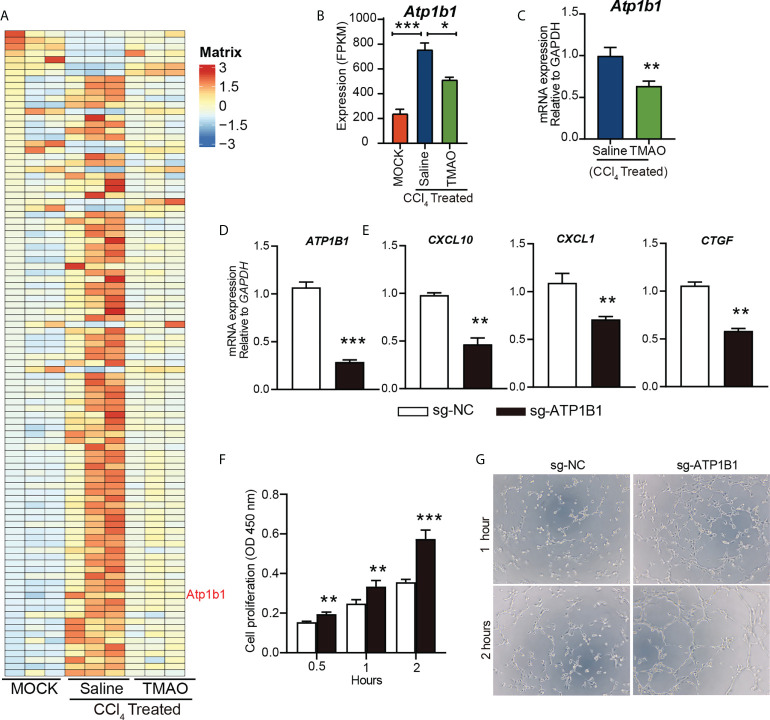
TMAO inhibits the expression of ATP1B1 in vascular endothelial cells **(A-B)** Heatmap depicting the differential gene expression levels in endothelial cells **(A)**. FPKM levels of ATP1B1 **(B, C)** ATP1B1 expression levels decreased after TMAO treatment. **(D)** Knockdown of ATP1B1 with CRISPR–Cas9 in HUVECs. **(E)** mRNA levels of CXCL10, CXCL1 and CTGF in HUVECs treated with sg-ATP1B1. **(F)** The effects of ATP1B1 on cell proliferation in HUVECs by CCK8. **(G)** The effects of ATP1B1 on vascular formation ability in HUVECs by tube formation assay. *p<0.05, **p<0.01, ***p<0.001.

Na+/K+-ATPase (NKA) has been proposed as a signal transducer involved in various pathobiological processes, including hepatocellular carcinoma (HCC) (33). We identified an enrichment of the ATP1B1 an astrocyte-specific isoform of the Na+/K+-ATPase transmembrane ionic pump, by RNA sequencing analysis of endothelial cells from a mouse liver fibrosis model (**Figure 5A**).

Interestingly, ATP1B1 expression levels were also decreased after TMAO treatment (**Figure 5B, C**), suggesting that ATP1B1 may be involved in the regulation of TMAO on endothelial cell function to attenuate liver fibrosis. To determine whether TMAO regulates endothelial cell function by targeting ATP1B1, we employed CRISPR–Cas9 to knock out the ATP1B1 gene and examined its function in endothelial cells. Efficacy for the knockout of ATP1B1 with CRISPR–Cas9 in HUVECs was evaluated with qPCR analysis, revealing a significant decrease (**Figure 5D**). Consistent with the effect of TMAO on endothelial gene regulation, the gene expression levels of profibrotic factors, including CXCL10, CXCL1 and CTGF, were significantly reduced in cells with ATP1B1 knockout in HUVECs (**Figure 5E**). Moreover, knockout of ATP1B1 promoted cell proliferation and tube formation of HUVECs (**Figures 5F, G**). These data suggest that the vascular protective effect of TMAO may be mediated by targeting endothelial cell ATP1B1.

### Blockage of ATP1B1 attenuates liver fibrosis

**Figure 6:**
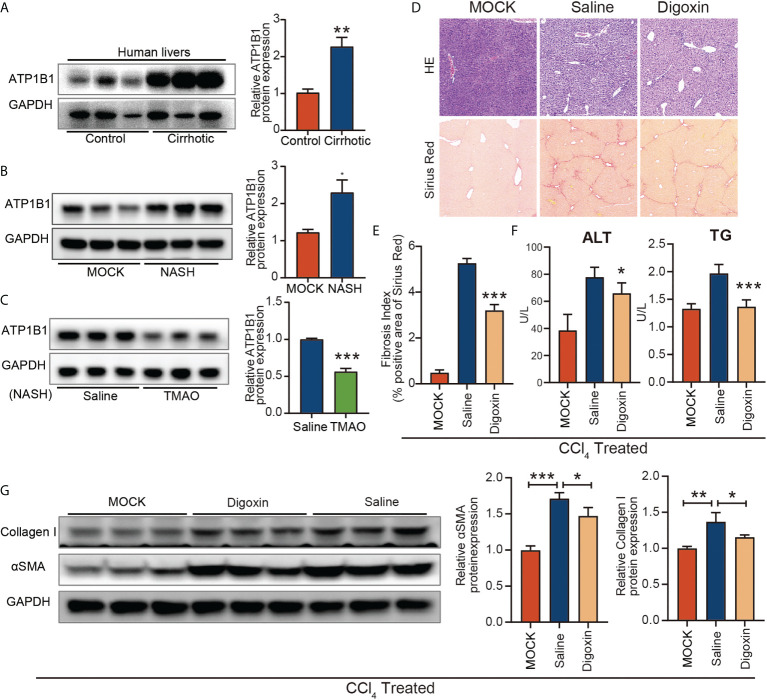
Blockage of ATP1B1 attenuates liver fibrosis **(A–C)** Immunoblots of ATP1B1 relative to GAPDH protein from NASH confirmed human and mouse samples **(A, B)**. Immunoblot analysis of ATP1B1 protein in NASH-TMAO mice **(C, D–E)** Digoxin reduces liver fibrosis by histological analysis of Sirius Red-stained sections. **(F)** Digoxin prevents hepatocellular damage as measured by the serum levels of ALT and TG. **(G)** Western blot of Collagen 1 and αSMA. *p<0.05, **p<0.01, ***p<0.001.

To investigate the role of ATP1B1 in liver fibrosis, we first examined the changes in ATP1B1 protein levels in patients with cirrhosis and mice with liver fibrosis. Western blotting showed that hepatic ATP1B1 protein levels were upregulated in both cirrhotic patients and NASH mice (**Figures 6A, B**). Moreover, the aberrant upregulation of ATP1B1 protein in fibrotic livers could be reversed by injection of TMAO (**Figure 6C**). These findings suggest that the aberrant upregulation of ATP1B1 may induce liver fibrosis. We then examined the effect of ATP1B1 blockade by the ATP1B1 inhibitor glycoside digoxin, a selective inhibitor of ATP1B1, on liver fibrosis. Histopathological analysis with Sirius red revealed a significant decrease in the acute CCl_4_ model after the intraperitoneal injection of 0.5 mg/kg digoxin relative to the control vehicle (**Figure 6D**). Western blot analysis of collagen 1 and αSMA showed that liver fibrosis was significantly attenuated after digoxin treatment. Liver function was also ameliorated after digoxin treatment, as shown by the downregulation of serum ALT and TG levels (**Figures 6F, G**). Collectively, these findings demonstrate that blockade of ATP1B1 halts disease progression in liver fibrosis mice and preserves liver function.

## Discussion

Gut microbial and microbial metabolite alterations contribute to the onset and progression of nonalcoholic fatty liver disease (34–36). TMAO is a circulating metabolite produced as a direct result of microbial degradation of dietary methylamines in the intestinal tract, which is associated with NASH, but correlation does not equate with causation. Our results shed new light and elucidate the mechanism underlying the roles of TMAO in NASH. We found that TMAO supplementation decreased liver fibrosis and improved liver function in an acute injury model and NASH model. It also restored the diversity of gut flora in a mouse NASH model. Zhao et al. had a similar report that TMAO was beneficial to the improvement of the structure of gut microbiota in a rat model induced by high-fat high-cholesterol (HFHC) diet feeding (37). It is worth mentioning that the mouse NASH model induced by Western diet and CCl_4_ is more advantageous in mimicking the histological, immunological, and transcriptomic features of human NASH than the HFHC model used in the previous study (37, 38), By using the mouse NASH model, we found the effect of TMAO on protecting the integrity of hepatic sinusoidal endothelium and identified the ATP1B1 instead of the canonical targets and pathways, revealing a new insight into microbiota-metabolite-vascular microenvironment crosstalk.

One of the most important findings of this study is the identification of an important role of TMAO in vascular endothelial cells (ECs). ECs are distributed in virtually all organs and modulate diverse pathophysiological functions (39). After tissue injury, ECs supply instructive paracrine/angiocrine factors to induce regeneration of adjacent parenchymal cells (40, 41). Moreover, the proliferation and vasculogenesis of ECs guarantee tissue injury repair. As shown in **Figures 4A, B**, injection of TMAO significantly increased the number of endothelial cells in TMAO-treated mice. Similar to our results, TMAO enhanced blood–brain barrier (BBB) integrity and protected it from inflammatory insult (26). In a previous study, it was reported that TMAO promotes liver steatosis in a mouse model of NAFLD (42). The difference in the effect of TMAO between this previous study and our study may be due to the following reasons. Firstly, the different dose of TMAO were used. The dose of TMAO used in the previous study was 400 mg/kg daily, which is 5 to 6 times higher than the dose of TMAO (75 mg/kg daily) used in the present study. Secondly, the different animal models were used in two studies. Unlike the NAFLD model induced by administration of high fat and cholesterol, our mouse NASH model by using a WD diet+CCl_4_ exhibits rapid progression of advanced fibrosis and HCC and perfectly mimics the characteristics of human NASH diseases. TEM and SEM analysis revealed that the basement membrane was reduced and fenestrations were increased in LSECs of TMAO-treated mice. Our findings demonstrate that injection of TMAO protects liver vascular function. Liver endothelial cells are mainly composed of LSECs, and normal LSECs can maintain hepatic stellate cell (HSC) in a resting state, however, LSECs lose the ability to control the resting state of HSC in chronic liver injury (30). Therefore, how to protect the integrity of LSEC is very important to improve liver fibrosis.

However, we do not know the exact mechanism of action of TMAO in endothelial cells. We identified an enrichment of the ATP1B1, an astrocyte-specific isoform of the Na^+^/K^+^-ATPase (NKA) transmembrane ionic pump, by RNA sequencing analysis of endothelial cells from a mouse liver fibrosis model (**Figure 5A**). The ion transporter NKA is a transmembrane protein that transports Na^+^ and K^+^ across cell membranes (43), which is essential for the cellular electrochemical gradient (44), ion homeostasis (45) and cell adhesion (46). Functional NKA consists of a subunits and b subunits. To date, 4 NKA a-subunits (a1, a2, a3, and a4) and 4 b-subunits (b1, b2, b3, and b4) have been identified. Abnormal NKA can lead to a variety of diseases, including hypokalemic periodic paralysis and CNS symptoms (47), cardiovascular disorders (48), atherosclerosis (49), Alzheimer’s disease (50), and hepatocellular carcinoma (HCC) (33). ATP1B1 is downregulated in human epithelial cancer cells (51–53). Shibuya et al. (54)and Li et al. (55) noted that ATP1A3 overexpression in HCC is related to the antitumor activity of bufalin. ATP1B3 and ATP1B1 were also significantly upregulated in HCC (33). However, the role ATP1B1 plays in the pathophysiology of NASH remains largely unknown. We first validated ATP1B1 at the protein level in NASH confirmed human and mouse samples by immunoblotting analysis, which increased in NASH and reduced in NASH-TMAO mice. It also occurred in endothelial cells of liver fibrosis. Next, I demonstrated the function of ATP1B1 both *in vivo* and *in vitro*. In HUVECs, ATP1B1 was knocked down with CRISPR–Cas9. To further elucidate the anti-inflammatory effects of ATP1B1 inhibition, we analyzed the critical inflammatory chemokines and cytokines CXCL10 and CXCL1 and the fibrosis factor CTGF. Our analysis revealed that these inflammatory and fibrotic factors were decreased in HUVECs with knockdown of ATP1B1. Proliferation and tube formation of HUVECs were used to assess the effect on vascular function. Compared with sg-NC, the knockdown of ATP1B1—sg-ATP1B1 showed better cell proliferation and vascular formation ability in HUVECs. These data confirm that the vascular protective effects were mediated by targeting ATP1B1 in endothelial cells. Meanwhile, *in vivo* animal experiments demonstrate that pharmacological inhibition with digoxin, a selective inhibitor of ATP1B1, halts disease progression in liver fibrosis mice and preserves liver function.

There are limitations to the current study. The contribution of an improved gut microbial profile to the attenuation of WD diet+CCl_4_-induced steatohepatitis by TMAO treatment was not fully illuminated. There are limitations to the current study. The contribution of an improved gut microbial profile to the attenuation of WD diet+CCl_4_-induced steatohepatitis by TMAO treatment was not fully illuminated. It has been reported that TMAO inhibits the synthesis of bile acids which disrupt the growth of intestinal flora (16). However, more investigations concerning the effect of TMAO on gut microbiota and metabolic consequences are needed. For example, the gut microbiota converts TMAO to TMA, and this TMA is reoxidized to TMAO, in line with the process of metabolic retroversion (56), and metabolic retroconversion of TMAO may be protective.

In summary, we have provided evidence that the gut microbial metabolite TMAO restores the diversity of gut flora, reduces liver fibrosis, and protects murine vascular function. We identified that the upregulation of ATP1B1 in ECs regulates an inflammatory response and vascular function that contributes to liver fibrosis in NASH mice. We provide evidence that inhibiting ATP1B1 might be effective for treating nonalcoholic steatohepatitis. Therefore, the present results reveal new insight into microbiota-metabolite-vascular microenvironment crosstalk.

## Data availability statement

The datasets presented in this study can be found in online repositories. The names of the repository/repositories and accession number(s) can be found in the article/**Supplementary Material**.

## Ethics statement

The studies involving human participants were reviewed and approved by the Ethical Committee of Biobank Center Related Hospitals and West China Hospital. The patients/participants provided their written informed consent to participate in this study. The animal study was reviewed and approved by the Institutional Animal Care and Use Committee of West China Second University Hospital, Sichuan University. Written informed consent was obtained from the individual(s) for the publication of any potentially identifiable images or data included in this article.

## Author contributions

DZ and JZ designed and performed the experiments. CX and CM analyzed the data and revised the manuscript. B-SD conceived the project, designed, and supervised the experiments. DZ, CM, and B-SD wrote the manuscript with input from all the authors. All authors contributed to the article, read, and approved the final manuscript.

## Funding

This work was supported by the Key Research and Development Program focused on Stem Cell and Translational Research (2016YFA0101600), National Science Foundation of China (91639117, 81941007, 81925005, 81722005, and 91839301), Sichuan Science and Technology Program (2019JDJQ0030, 2019YJ0059), the Fundamental Research Funds for the Central Universities, and Sichuan University postdoctoral interdisciplinary Innovation Fund.

## Conflict of interest

The authors declare that the research was conducted in the absence of any commercial or financial relationships that could be construed as a potential conflict of interest.

## Publisher’s note

All claims expressed in this article are solely those of the authors and do not necessarily represent those of their affiliated organizations, or those of the publisher, the editors and the reviewers. Any product that may be evaluated in this article, or claim that may be made by its manufacturer, is not guaranteed or endorsed by the publisher.
